# Endocrine Autoimmunity and Inflammatory Signatures in Pediatric Celiac Disease: Context-Dependent Patterns

**DOI:** 10.3390/diagnostics16091330

**Published:** 2026-04-28

**Authors:** Marta Greco, Maria Mirabelli, Roberta Misiti, Francesco Dragone, Annalidia Donato, Denise Casella, Antonio Torchia, Daniela Concolino, Antonio Brunetti, Daniela P. Foti

**Affiliations:** 1 Department of Health Sciences, University “Magna Græcia” of Catanzaro, 88100 Catanzaro, Italy; 2 Operative Unit of Clinical Pathology, “R. Dulbecco” Hospital, 88100 Catanzaro, Italy; 3 Department of Experimental and Clinical Medicine, University “Magna Græcia” of Catanzaro, 88100 Catanzaro, Italy

**Keywords:** celiac disease, children, thyroid autoimmunity, islet autoantibodies, cytokines

## Abstract

**Background:** Celiac disease (CD) is frequently associated with autoimmune disorders such as Hashimoto’s thyroiditis and type 1 diabetes. Endocrine-specific autoantibodies may emerge during the course of CD, but their true prevalence and clinical relevance in children remain unclear. This study evaluated endocrine autoantibodies and inflammatory profiles in pediatric CD to inform a more tailored diagnostic approach. **Methods:** In this retrospective cross-sectional study, 240 consecutive children referred to a tertiary center for suspected CD and/or autoimmune endocrine disorders were included. CD was diagnosed according to ESPGHAN criteria. Laboratory evaluation comprised blood counts, metabolic parameters, thyroid function tests, thyroid autoantibodies (anti-TPO, anti-Tg), and type 1 diabetes-related autoantibodies (GAD, IA-2, ZnT8). A subgroup of children with CD (*n* = 28) underwent exploratory multiplex cytokine analysis. **Results:** Children with CD were slightly older and more often female than controls. Platelet counts were modestly lower in CD, while other hematologic parameters were similar. Thyroid autoimmunity prevalence did not differ significantly (anti-TPO: 2.7% in CD vs. 5.4% in controls; *p* = 0.348), and antibody titers and TSH levels were comparable. Anti-TPO positivity was associated with older age (*p* = 0.038), independent of CD status. Islet autoantibodies were similarly distributed between groups. Cytokine levels were not associated with tTG-IgA status; however, girls with CD showed higher IL-2, IL-4, and IL-10 levels than boys (all *p* < 0.05), with a trend toward higher IL-1α. **Conclusions:** In this pediatric cohort enriched for immune and endocrine concerns, CD was not linked to increased thyroid or pancreatic autoimmunity. Distinct sex-related differences in inflammatory profiles were observed, suggesting distinct immune patterns in girls with CD. These findings support a clinically driven rather than routine approach to endocrine autoantibody screening and warrant further studies on cytokine-based immune stratification.

## 1. Introduction

Initially described as a cause of intestinal malabsorption reversible with dietary gluten withdrawal [1], celiac disease (CD) is now recognized as a systemic immune-mediated disorder, rather than a condition confined to the intestine [2]. Classic gastrointestinal manifestations, including diarrhea and abdominal pain [3], may coexist with a broad spectrum of extraintestinal features such as headache [4], anemia [5], infertility [6], and elevated liver enzyme levels [7]. These diverse clinical presentations reflect a complex immune-mediated pathophysiology extending beyond the intestinal mucosa.

The underlying mechanism involves activation of the adaptive immune response, driven by intestinal mucosal T lymphocytes, and the production of autoantibodies against tissue transglutaminase (tTG), triggered by gluten exposure in genetically predisposed individuals. In such subjects, deamidated gliadin peptides are presented by antigen-presenting cells expressing HLA-DQ2 or HLA-DQ8 molecules, initiating and sustaining the autoimmune cascade [8]. Genetic susceptibility, particularly carriage of HLA-DQ2 and/or HLA-DQ8 haplotypes, represents a major risk factor for CD development. Although these genotypes are present in the vast majority of affected individuals, they are not sufficient alone to induce disease [9]. Environmental factors, including timing and quantity of gluten introduction, mode of delivery, breastfeeding practices [10,11,12], and infections [8], also contribute to disease onset, together with additional immunological determinants that remain incompletely understood.

Globally, CD affects approximately 1% of the population, with significant geographic variability [13], and shows higher prevalence in females and in pediatric populations [13,14]. In children, clinical presentation is atypical or nonspecific in up to 95% of cases, frequently resulting in delayed or missed diagnosis [15].

Over the past three decades, pediatric diagnoses have increased due to improved screening strategies [13,14] and widespread use of highly sensitive and specific serological assays, enabling broader detection across populations [15]. Refinement of serological testing, including anti-tTG, deamidated gliadin peptide, and anti-endomysial antibodies, has allowed for diagnosis in many pediatric cases without intestinal biopsy when antibody titers exceed defined thresholds, in accordance with ESPGHAN guidelines [9].

Several clinical conditions increase susceptibility to CD, including genetic syndromes, such as Down, Turner, and Williams syndromes [16]. Moreover, CD frequently coexists with other autoimmune [17] and endocrine disorders [18], with an overall risk approximately three times higher than in the general population [19,20]. A bidirectional association exists between CD and autoimmune endocrine conditions. Increased incidence is observed in patients with type 1 diabetes mellitus and Hashimoto’s thyroiditis [2], while conversely, CD prevalence is elevated among individuals with autoimmune endocrinopathies, although estimates vary widely across populations [21,22]. These disorders likely share overlapping genetic predisposition, environmental triggers, and immune–endocrine regulatory mechanisms that remain incompletely elucidated [23,24].

Among autoimmune comorbidities, coexistence of CD and type 1 diabetes occurs in approximately 8% of pediatric cases [23]. However, the clinical and immunological phenotype of patients with a higher autoimmune burden remains insufficiently characterized. Earlier onset of diabetes (around 3–4 years of age) has been reported in children with concomitant CD, compared with approximately 7 years in those with isolated type 1 diabetes [22,23,24,25], suggesting possible amplification of immune dysregulation. Whether this enhanced autoimmune susceptibility correlates with active celiac serology (e.g., elevated tTG-IgA titers or symptomatic disease) or may be attenuated by dietary treatment remains unclear [25]. Early detection of islet autoantibodies in at-risk children could enable closer metabolic surveillance and reduce the risk of severe diabetes onset with ketoacidosis [26]. However, the clinical utility and optimal timing of systematic autoimmune screening in CD remain debated, particularly given variable prevalence in control populations and the possibility of transient, clinically insignificant autoantibody positivity [27].

The association between CD and Hashimoto’s thyroiditis presents additional challenges. Meta-analyses indicate that 4–6% of individuals with CD develop autoimmune thyroid disease, corresponding to an approximately fourfold increased risk compared with the general population [21], with a particularly high likelihood of progression hypothyroidism during childhood [28]. Hashimoto’s thyroiditis is more prevalent in adults than in children and adolescents and shows marked female predominance [21]. However, the prevalence and clinical impact of thyroid autoimmunity in pediatric CD remain variably reported, especially in clinically heterogeneous populations of children. Age-related patterns of autoimmune clustering have been described, with arthropathies and connective tissue diseases predominating in adults and type 1 diabetes and CD being more common in pediatric populations [24].

The interaction between active CD and thyroid autoimmunity remains an area of active investigation. Cross-reactivity of anti-tTG antibodies with thyroid antigens has been proposed [29], and iron deficiency, frequent in untreated CD, has been implicated in thyroid dysfunction [25]. Furthermore, recent evidence in adults with Hashimoto’s thyroiditis indicates that concomitant gastrointestinal-related autoantibodies are associated with a broader inflammatory signature, including elevated IL-1α levels, particularly in females [30]. Barrier epithelia, including the gastrointestinal tract, are enriched in IL-1α, an alarmin released upon cellular injury that promotes local inflammation and systemic immune amplification via T-cell activation [30]. Still, these mechanisms have been described predominantly in adult populations, and their relevance in pediatric CD remains incompletely established.

Taken together, these observations suggest that immune–endocrine interactions in CD are complex and may vary according to age and clinical context. Despite growing interest in this field, the prevalence and clinical significance of endocrine-related autoantibodies in children remain poorly defined, particularly in real-world clinical settings enriched for immune and endocrine conditions. In addition, data on inflammatory signatures in pediatric CD are limited, with scarce information on sex-related differences. This gap is clinically relevant, as a clearer characterization of autoimmune and inflammatory patterns may improve risk stratification and support more targeted screening strategies in pediatric CD. In this context, the present study aimed to evaluate the prevalence and clinical relevance of endocrine-associated autoantibodies in a real-world cohort of pediatric patients with CD compared with a heterogeneous population of non-celiac inpatients and to explore associated inflammatory profiles, with particular attention to sex-related immune differences.

## 2. Materials and Methods

### 2.1. Study Design and Population

This retrospective observational cross-sectional study was conducted at the Clinical Pathology Unit of the University Hospital “Renato Dulbecco” in Catanzaro, Italy. Clinical and laboratory data routinely generated during standard diagnostic evaluation were collected from 240 consecutive pediatric patients referred from the Pediatric Unit for assessment of CD and/or autoimmune endocrine-related laboratory testing between September 2024 and February 2026. The study population reflects a real-world clinical setting, as most subjects were inpatients admitted for conditions unrelated to CD but frequently requiring immunological or endocrine evaluation, rather than a cohort of healthy controls. Patients were classified into CD and non-celiac groups based on the clinical diagnosis at admission and supported by prior laboratory evaluation. In the CD group, patients had a previous or current diagnosis of celiac disease made by the treating pediatricians in accordance with ESPGHAN guidelines. Specifically, diagnosis was established on the basis of celiac serology, including tTG-IgA and, when appropriate, anti-endomysial antibodies, with duodenal biopsy performed only when clinically indicated according to guideline-based diagnostic pathways. The tTG-IgA values reported in the present study refer to measurements obtained at the time of enrollment, which in some cases corresponded to follow-up assessments, and were therefore used to define current serological status (tTG-IgA-positive vs. tTG-IgA-negative), rather than to establish the original diagnosis of CD. Consequently, some patients with an established diagnosis of CD were tTG-IgA-negative at study evaluation, likely reflecting follow-up after gluten withdrawal or serological normalization. Exclusion criteria included severe anemia or hematologic disorders, evidence of acute infection (defined as leukocytosis >15 × 10^3^/µL), and uncontrolled thyroid dysfunction with hormone levels outside age-adjusted reference ranges. All laboratory parameters available from routine clinical practice were retrospectively extracted, including thyroid function tests, thyroid autoantibodies, complete blood counts, metabolic parameters, and type 1 diabetes-related autoantibodies. Laboratory investigations were performed according to clinical indications, resulting in variable availability of specific tests across subjects.

### 2.2. Routine Laboratory Measurements

Routine laboratory and immunological analyses were performed in the fasting state as part of standard patient care. Thyroid-stimulating hormone (TSH), anti-thyroid peroxidase antibodies (anti-TPO), and anti-thyroglobulin antibodies (anti-Tg) were measured using chemiluminescent immunoassays on the ADVIA Centaur^®^ system (Siemens Healthcare Diagnostics, Camberley, UK), applying manufacturer-recommended cut-off values (TSH: 0.55–4.78 mIU/mL; anti-TPO: >60 IU/mL; anti-Tg: >4.5 IU/mL). Serum insulin and C-peptide concentrations were determined by chemiluminescent immunoassays on the same platform, while plasma glucose levels were measured using enzymatic assays on the Cobas Pro analyzer (Roche Diagnostics, Rotkreuz, Switzerland). Complete blood counts were obtained using the ADVIA 2120i hematology analyzer (Siemens Healthcare Diagnostics). Celiac serology included anti-tissue transglutaminase IgA antibodies measured by fluoroenzyme immunoassay on the Phadia 250 system (Thermo Fisher Scientific, Waltham, MA, USA), considering a positivity cut-off of >10 U/mL. Type 1 diabetes-related autoantibodies, including glutamic acid decarboxylase (GAD), insulinoma-associated antigen-2 (IA-2), and zinc transporter 8 (ZnT8), were assessed using commercially available enzyme-linked immunosorbent assay (ELISA) kits (Euroimmun Medizinische Labordiagnostika AG, Lübeck, Germany), according to manufacturer instructions. The following cut-off values were applied: GAD, >10 IU/mL; IA-2, >10 IU/mL; and ZnT8 >15 IU/mL. In ELISA tests for GAD, IA2, and ZnT8, intra-assay coefficients of variation (CVs) were <9%, <4% and <9%, and inter-assay CVs were <6%, <5% and <10%, respectively. All assays were performed within the same hospital laboratory unit to ensure analytical consistency. The measurements were carried out following standardized procedures and were routinely monitored through internal quality control programs and participation in external quality assessment (EQA) schemes.

### 2.3. Exploratory Cytokine Profiling

**Figure 1:**
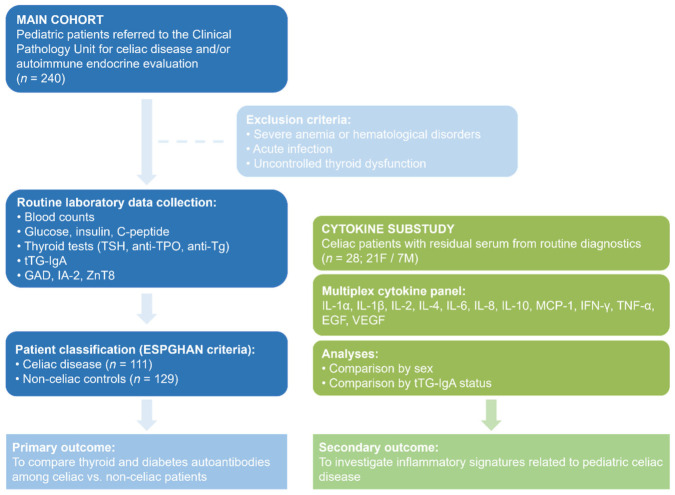
Flowchart of the study.

To investigate inflammatory patterns in pediatric CD, an exploratory cytokine substudy was conducted in a subset of patients with available residual serum samples from routine diagnostics. Leftover sera from 28 pediatric patients with CD (21 females and 7 males), otherwise destined for disposal, were anonymized and used for cytokine analysis. Serum concentrations of IL-1α, IL-1β, IL-2, IL-4, IL-6, IL-8, IL-10, MCP-1, IFN-γ, TNF-α, EGF, and VEGF were simultaneously quantified using the Cytokine Array I biochip on the Evidence Investigator analyzer (Randox Laboratories, Crumlin, UK), following manufacturer protocols. For cytokine measurements, both intra-assay and inter-assay CVs were <10%. Figure 1 illustrates the flowchart of the study.

### 2.4. Statistical Analysis

Continuous variables were expressed as the median and interquartile range (IQR) and compared between groups using the Mann–Whitney U test. Categorical variables were expressed as counts and percentages and compared using the chi-square test or Fisher’s exact test, as appropriate. Comparisons were performed between patients with and without CD, between patients with and without anti-thyroid peroxidase (anti-TPO) antibody positivity, and between patients with and without tTG-IgA positivity. Islet autoimmunity was defined as the presence of at least one positive type 1 diabetes-related autoantibody (GAD, IA-2, or ZnT8). Due to the retrospective design and the real-world clinical setting, not all laboratory parameters were available for all children. Therefore, analyses were conducted using a complete-case approach for each variable. No imputation was performed, and denominators are reported accordingly. Although this approach may limit comparability across subgroups, it reflects routine clinical practice and avoids introducing assumptions related to missing data. Cytokine levels were analyzed only within the subgroup of CD patients with available cytokine measurements and were compared according to sex and tTG-IgA status. A significance level of 0.05 was set for all analyses. Data were analyzed with JASP Graphical Statistical Software Version 0.17.2 (University of Amsterdam, Amsterdam, The Netherlands) based on R Stats packages.

## 3. Results

### 3.1. Clinical and Immunological Characteristics of Celiac and Non-Celiac Patients

A total of 240 pediatric patients were included in the analysis, of whom 111 had CD and 129 were non-celiac inpatients referred to the same Pediatric Unit during the enrollment period for conditions unrelated to CD, who underwent immunological and/or endocrine assessments (Table 1). Within the control group, congenital hypothyroidism/other endocrine conditions, allergies, and genetic syndromes accounted for a substantial proportion of admissions. Infectious diseases (mild or self-limiting; i.e., viral or parasitic, without acute leukocytosis) were infrequent in this inpatient pediatric population and did not differ significantly between groups. Patients with CD were slightly older than non-celiac patients (median age 10.5 [IQR 7.0–14.0] vs. 9.0 [6.0–13.0] years, *p* = 0.042). Among hematological parameters, platelet counts were significantly lower in celiac patients than in non-celiac patients (276.5 [237.8–334.0] vs. 305.0 [249.0–365.0] × 10^9^/L, *p* = 0.020), whereas white blood cell count, red blood cell count, hemoglobin, MCV, RDW, and other hematologic indices did not differ significantly.

Thyroid function and thyroid autoimmunity markers did not differ between groups in terms of anti-TPO, anti-Tg, or TSH levels. Anti-TPO antibody positivity was observed in 5.4% of non-celiac patients and 2.7% of celiac patients, without a statistically significant difference (*p* = 0.348). Among celiac patients, 30.6% had detectable tTG-IgA positivity at the time of enrollment and laboratory screening, indicating that current seropositivity was present only in a subset of children with an already established diagnosis of CD. Islet autoantibody positivity, defined as the presence of at least one type 1 diabetes-related autoantibody (GAD, IA-2, or ZnT8), did not differ between groups when calculated using only patients tested for these autoantibodies (5/59 [8.5%] in non-celiac vs. 4/82 [4.9%] in celiac patients, *p* = 1.000). Sex distribution differed significantly between groups, with a lower proportion of males among celiac patients than among non-celiac patients (29.7% vs. 48.8%, *p* = 0.004), consistent with the reported female predominance of CD.

To further explore the clinical relevance of type 1 diabetes-related autoantibody positivity, metabolic parameters were compared between autoantibody-positive and -negative patients. Autoantibody-positive patients showed slightly higher fasting glucose levels compared with autoantibody-negative patients (83.0 [81.8–83.8] vs. 76.0 [72.0–80.0] mg/dL, *p* = 0.037), while fasting C-peptide levels did not differ significantly between groups.

As shown in Table 2, patients with anti-TPO positivity were older than anti-TPO-negative patients (median age 14.0 [12.0–14.0] vs. 10.0 [6.0–13.3] years, *p* = 0.038). As expected, anti-TPO-positive patients had markedly higher anti-TPO and anti-Tg levels (both *p* < 0.001). No significant differences were observed between anti-TPO-positive and anti-TPO-negative patients with respect to TSH, hematological parameters, glucose metabolism markers, or diabetes-related autoantibody titers. Congenital hypothyroidism or other endocrine diseases were more frequent in anti-TPO-positive patients (30.0% vs. 7.9%, *p* = 0.048), and genetic syndromes were also more prevalent (30.0% vs. 4.4%, *p* = 0.012). The prevalence of CD did not differ significantly between groups. Islet autoantibody positivity, calculated among tested patients only, was observed in 9/138 (6.5%) anti-TPO-negative patients and in none of the anti-TPO-positive patients (0/3), with no statistically significant difference (*p* = 1.000). Sex distribution did not differ significantly between groups (41.2% vs. 20.0% males, *p* = 0.323), although a female predominance was observed among anti-TPO-positive patients (Table 2).

### 3.2. Comparison Between tTG-IgA-Negative and tTG-IgA-Positive Patients with Celiac Disease and Influence of Sex on Inflammatory Markers

Among patients with an established diagnosis of CD, 34 were tTG-IgA-positive and 68 were tTG-IgA-negative at the time of study evaluation (Table 3). Thus, in this analysis, tTG-IgA status reflects serological activity at the time of laboratory reassessment and not the criterion by which CD had originally been diagnosed. No significant differences were observed between the two groups in terms of sex distribution, age, hematologic parameters, thyroid function, or thyroid autoantibody levels, although anti-TPO levels tended to be higher in tTG-IgA-positive patients (*p* = 0.051). As expected, tTG-IgA titers were significantly higher in the tTG-IgA-positive group (*p* < 0.001). Markers of glucose metabolism and diabetes-related autoantibodies did not differ significantly between groups. Clinical comorbidities, including congenital hypothyroidism/other endocrine conditions, allergies, and genetic syndromes, were rare and showed no significant differences. Islet autoantibody positivity, calculated using only tested patients, was observed in 4/64 (6.3%) tTG-IgA-negative patients and in none of the tTG-IgA-positive patients (0/11), without a statistically significant difference (*p* = 0.298). Cytokine concentrations did not differ significantly between tTG-IgA-positive and tTG-IgA-negative celiac patients for any of the analyzed cytokines. However, when cytokines were compared by sex among celiac patients with available cytokine measurements (females *n* = 21; males *n* = 7) (Table 4), female patients showed significantly higher levels of IL-2, IL-4, and IL-10 (all *p* < 0.05). No significant sex-related differences were observed for IL-6, IL-8, VEGF, IFN-γ, TNF-α, IL-1α, IL-1β, MCP-1, or EGF, while IL-1α showed a borderline trend (*p* = 0.059).

To assess whether cytokine differences were associated with variations in autoimmune burden, autoantibody titers were further analyzed by sex within the same subgroup of celiac patients with cytokine data (Table 5). No significant sex-related differences were observed in anti-TPO or anti-Tg titers. A borderline higher tTG-IgA titer was observed in female patients compared with male patients (56.0 [1.0–128.0] vs. 0.30 [0.20–0.90], *p* = 0.051).

## 4. Discussion

In this cross-sectional pediatric cohort, we investigated the clinical, immunological, and inflammatory profiles of patients with CD compared with non-celiac pediatric inpatients, with particular attention to thyroid autoimmunity, pancreatic autoantibodies, and cytokine patterns. CD was associated with modest differences in age and platelet count but not with an increased prevalence of thyroid or pancreatic autoimmunity compared with a heterogeneous inpatient pediatric population characterized by immune-related conditions. The slightly lower platelet count observed in celiac patients, although small in magnitude, aligns with existing evidence that hematologic alterations in CD may reflect autoimmune mechanisms, nutritional deficiencies, and possible splenic or hepatic involvement, contributing to either thrombocytopenia or thrombocytosis [31].

Although autoimmune thyroid disease is frequently reported in association with CD [21], we did not observe higher anti-TPO positivity or elevated thyroid autoantibody titers among celiac patients compared with controls. In pediatric settings where non-celiac inpatients often present with immune-mediated or endocrine disorders, the relative burden of thyroid autoimmunity may therefore not be uniquely increased in CD. Several conditions sharing genetic susceptibility, such as Down syndrome, Turner syndrome, and polyautoimmune syndromes, are independently associated with Hashimoto thyroiditis [32,33,34]. Environmental factors, including nutritional deficiencies (e.g., vitamin D, selenium, iodine), infections, intestinal dysbiosis, and environmental pollutants, have also been implicated as potential triggers of thyroid autoimmunity [33,34,35,36]. Additionally, childhood obesity has been linked to autoimmune thyroid diseases, possibly through increased leptin-mediated immune modulation, microbiome alterations, and chronic low-grade inflammation [37]. These overlapping risk factors may partially explain the comparable prevalence observed in our cohort.

Similarly, the prevalence of diabetes-related autoantibodies did not differ between celiac and non-celiac patients when analysis was restricted to those who underwent testing. This observation suggests that subclinical pancreatic autoimmunity may occur across pediatric populations with diverse clinical backgrounds and may not be specifically enriched in CD. Although previous studies have reported increased type 1 diabetes autoantibodies in celiac cohorts, many relied on general population controls or incomplete autoantibody screening, potentially overestimating disease-specific associations [38].

The expected female predominance of CD was confirmed in our cohort, consistent with the broader epidemiology of autoimmune disorders. Beyond prevalence, sex differences extended to inflammatory profiles. While cytokine levels did not differ according to tTG-IgA status among celiac patients, females with available cytokine measurements exhibited significantly higher levels of IL-2, IL-4, and IL-10 compared with males. These findings align with evidence that sex-related differences in immune responses are detectable in childhood, although typically less pronounced than in adulthood, when hormonal influences further modulate immune function [39,40]. Increased IL-2 and IL-4 production in females has been associated with enhanced T-cell activation and immune polarization [41,42], while elevated IL-10 levels may reflect a more robust immunoregulatory response, potentially influenced by sex-related genetic and epigenetic mechanisms [43,44].

A similar trend toward higher IL-1α levels in girls may indicate sex-related differences in mucosal immune activation. Experimental and clinical data suggest that female intestinal mucosa may exhibit higher baseline activation of inflammatory pathways, including IL-1 signaling [45]. Although our findings suggest the possibility of a sex-specific inflammatory signature in pediatric CD, the limited sample size, particularly among males, warrants cautious interpretation. Moreover, the absence of an association between IL-1α and tTG-IgA levels highlights the complex and potentially asynchronous regulation of mucosal inflammation and autoantibody responses [46]. Together, these findings support the concept that serological markers and cytokine profiles reflect complementary but distinct aspects of disease biology.

The pathogenesis of CD involves coordinated activation of both Th1- and Th2-mediated immune responses [8]. Gluten exposure activates CD4+ lymphocytes within the intestinal mucosa, promoting a Th1-polarized response characterized by TNF, IFN-γ, and IL-2 production, which drives epithelial damage [46].

IL-2 release occurs rapidly after gluten stimulation and correlates with the degree of mucosal injury and clinical symptom severity, serving as a marker of T-cell activation [47]. Concurrent activation of the Th2 axis, mediated in part by IL-4, supports B-cell activation and antibody production [46,48]. IL-10, produced primarily by regulatory T cells but also by Th2 lymphocytes and antigen-presenting cells, plays an essential role in limiting excessive inflammation and preserving intestinal homeostasis. Experimental models confirm that IL-10 deficiency results in severe intestinal inflammation [49,50].

Beyond their individual effects, these cytokines exert synergistic action that shape the intestinal inflammatory milieu. Integrated IL-2 and IL-4 signaling can enhance downstream regulatory pathways, including IL-10 production and the expansion of regulatory T cells, thereby modulating effector responses [51]. Clinically, IL-2 release assays following gliadin stimulation have shown promise in identifying gluten-specific memory T cells, even in patients adhering to a gluten-free diet in whom conventional serological markers may be negative [52]. Circulating cytokine patterns may also provide insights into disease activity and mucosal damage severity [52]. The immunomodulatory properties of IL-10 further suggest potential therapeutic relevance in controlling intestinal inflammation [50].

Several limitations should be acknowledged. The relatively small number of patients with available cytokine measurements, particularly males, limits statistical power for sex-stratified analyses. The cross-sectional design precludes causal inference and does not allow for evaluation of longitudinal progression of autoantibody positivity. Furthermore, due to the real-world nature of the study, not all patients underwent comprehensive autoantibody testing. Despite these limitations, the study provides an integrated overview of autoimmune clustering and inflammatory patterns in pediatric CD within a clinically relevant inpatient population. Importantly, the non-celiac comparison group does not represent a healthy population but rather a heterogeneous cohort of pediatric inpatients frequently undergoing immunological or endocrine evaluation. This choice reflects real-world clinical practice but may have influenced the baseline prevalence of autoimmune markers, potentially attenuating differences between groups. Therefore, the absence of increased thyroid or pancreatic autoimmunity in CD should be interpreted with caution in light of the characteristics of the control population. In addition, the relatively small sample size in subgroup analyses, particularly for diabetes-related autoantibodies and cytokine profiling, may have limited statistical power and reduced the ability to detect subtle differences. The observed sex-related differences in cytokine levels should be considered exploratory and require confirmation in larger, adequately powered studies.

Overall, our findings suggest that pediatric CD does not uniformly confer an increased risk of concomitant thyroid or pancreatic autoimmunity when compared with other immune-related pediatric conditions. Thyroid autoimmunity appears to occur primarily in pubertal girls, whereas subclinical islet autoantibodies positivity did not display clinical correlates, for sex specificity, or association with celiac serology. Given that autoantibody positivity does not necessarily progress to overt autoimmune disease [38], systematic screening strategies in children with CD should be carefully considered. In light of heterogeneous international recommendations, a tailored approach, guided by clinical features and individual risk factors may help minimize unnecessary investigations and potential overdiagnosis while ensuring appropriate monitoring of high-risk patients.

## 5. Conclusions

CD often co-occurs with other autoimmune and endocrine conditions; however, laboratory evaluation should be guided by clinical context rather than indiscriminate screening. In our real-world cohort, the prevalence of endocrine-related autoantibodies in children with CD did not differ substantially from that observed in other pediatric clinical conditions requiring immunological or endocrine evaluation. Although these findings should be interpreted with caution, given the characteristics of the study population, they suggest that a generalized endocrine-related autoantibody screening strategy based solely on the diagnosis of CD may not be justified in the absence of specific clinical indications. Cytokine profiling represents a promising tool for investigating immune system dysregulation in CD and its associated autoimmune disorders, including type 1 diabetes and Hashimoto thyroiditis, but it remains unclear whether specific cytokine patterns may predict disease severity, the risk of autoimmune comorbidities, or the inflammatory response to treatment. Furthermore, cytokine signatures may potentially contribute to differential diagnosis in complex clinical settings, although this requires further validation. Larger studies, with inclusion of healthy control populations, and longitudinal designs are required to validate the clinical relevance of cytokine measurements and to define their role in routine pediatric practice.

## Figures and Tables

**Table 1:** Clinical and immunological characteristics of celiac and non-celiac patients.

Variable	Non-Celiac (*n* = 129)	Celiac (*n* = 111)	*p*-Value
Age (years)	9.00 [6.00–13.00]	10.50 [7.00–14.00]	0.042 *
Anti-TPO (U/mL)	33.10 [28.00–38.60]	30.90 [28.00–38.30]	0.375
Anti-Tg (IU/mL)	1.30 [1.30–1.30]	1.30 [1.30–1.30]	0.439
TSH (µIU/mL)	2.19 [1.50–3.00]	2.27 [1.65–2.86]	0.875
White blood cells (*n* × 10^3^/μL)	7.67 [6.60–8.98]	6.75 [5.80–9.53]	0.078
Red blood cells (*n* × 10^6^/μL)	4.89 [4.61–5.15]	4.87 [4.71–5.23]	0.487
Hemoglobin (g/dL)	12.90 [12.25–13.90]	13.20 [12.50–13.83]	0.407
MCV (fL)	80.90 [77.70–83.90]	81.90 [78.10–84.33]	0.377
RDW (%)	13.70 [13.20–14.40]	13.70 [13.00–14.20]	0.180
Platelets (*n* × 10^3^/μL)	305.00 [249.00–365.00]	276.50 [237.75–334.00]	0.020 *
GAD (IU/mL)	5.00 [5.00–5.00]	5.00 [5.00–5.00]	0.099
IA-2 (IU/mL)	10.00 [10.00–10.00]	10.00 [10.00–10.00]	0.153
ZnT8 (RU/mL)	10.00 [10.00–10.00]	10.00 [10.00–10.00]	0.318
Infectious diseases	4 (3.1%)	0 (0.0%)	0.126
Congenital hypothyroidism/endocrine disease	21 (16.3%)	1 (0.9%)	<0.001 *
Allergies	21 (16.3%)	0 (0.0%)	<0.001 *
Genetic syndromes	12 (9.3%)	1 (0.9%)	0.004 *
Anti-TPO positivity	7 (5.4%)	3 (2.7%)	0.348
tTG-IgA positivity ^§^	0 (0.0%)	34 (30.6%)	<0.001 *
Islet autoantibody positivity (GAD, IA-2 or ZnT8) ^‡^	5/59 (8.5%)	4/82 (4.9%)	1.000
Sex (male)	63 (48.8%)	33 (29.7%)	0.004 *

^‡^ Valid percentages for islet autoantibody positivity were calculated using the number of patients tested for type 1 diabetes-related autoantibodies (GAD, IA-2, or ZnT8) as the denominator. * denotes significant results. ^§^ tTG-IgA status refers to serology measured at the time of study evaluation and not necessarily at the time of CD diagnosis.

**Table 2:** Comparison between anti-TPO-negative and anti-TPO-positive patients.

Variable	Anti-TPO-Negative (*n* = 228)	Anti-TPO-Positive (*n* = 10)	*p*-Value
Age (years)	10.00 [6.00–13.25]	14.00 [12.00–14.00]	0.038 *
Anti-TPO (U/mL)	30.90 [28.00–38.02]	606.95 [268.70–1300.00]	<0.001 *
Anti-Tg (IU/mL)	1.30 [1.30–1.30]	2.30 [1.30–7.55]	<0.001 *
TSH (µIU/mL)	2.19 [1.55–2.92]	2.71 [2.08–3.97]	0.245
GAD (IU/mL)	5.00 [5.00–5.00]	5.00 [5.00–5.00]	0.782
IA-2 (IU/mL)	10.00 [10.00–10.00]	10.00 [10.00–10.00]	0.697
ZnT8 (RU/mL)	10.00 [10.00–10.00]	10.00 [10.00–10.00]	0.755
C-Peptide (ng/mL)	1.26 [0.79–1.65]	1.48 [1.25–1.70]	0.726
Glycemia (mg/dL)	76.00 [72.00–80.00]	80.00 [79.00–81.00]	0.312
Infectious diseases	4 (1.8%)	0 (0.0%)	1.000
Congenital hypothyroidism/endocrine disease	18 (7.9%)	3 (30.0%)	0.048 *
Allergies	21 (9.2%)	0 (0.0%)	0.607
Genetic syndromes	10 (4.4%)	3 (30.0%)	0.012 *
Celiac disease	106 (46.5%)	3 (30.0%)	0.351
Anti-Tg positivity	2 (0.9%)	4 (40.0%)	<0.001 *
tTG-IgA positivity	31 (13.6%)	1 (10.0%)	1.000
Islet autoantibody positivity (GAD, IA-2 or ZnT8) ^‡^	9/138 (6.5%)	0/3 (0.0%)	1.000
Sex (male)	94 (41.2%)	2 (20.0%)	0.323

^‡^ Valid percentages for islet autoantibody positivity were calculated using the number of patients tested for type 1 diabetes-related autoantibodies (GAD, IA-2, or ZnT8) as the denominator. * denotes significant results.

**Table 3:** Comparison between tTG-IgA-negative and tTG-IgA-positive celiac patients.

Variable	tTG-IgA-Negative (*n* = 68)	tTG-IgA-Positive (*n* = 34)	*p*-Value
Age (years)	11.00 [8.00–15.00]	10.00 [6.75–13.00]	0.214
Anti-TPO (U/mL)	30.05 [28.00–36.67]	36.20 [28.23–40.20]	0.051
Anti-Tg (IU/mL)	1.30 [1.30–1.30]	1.30 [1.30–1.30]	0.761
TSH (µIU/mL)	2.26 [1.65–2.74]	2.25 [1.66–2.79]	0.490
White blood cells (*n* × 10^3^/μL)	7.11 [5.67–9.51]	6.43 [5.77–9.74]	0.728
Red blood cells (*n* × 10^6^/μL)	4.86 [4.71–5.16]	4.88 [4.69–5.26]	0.911
Hemoglobin (g/dL)	13.20 [12.50–13.90]	13.00 [12.45–13.70]	0.528
MCV (fL)	82.20 [78.90–84.30]	79.70 [76.20–84.65]	0.521
RDW (%)	13.50 [12.90–14.20]	13.70 [13.35–14.65]	0.188
Platelets (*n* × 10^3^/μL)	275.00 [235.00–321.00]	294.00 [253.50–352.00]	0.336
tTG-IgA (IU/mL)	0.60 [0.10–1.62]	56.50 [19.75–128.00]	<0.001 *
GAD (IU/mL)	5.00 [5.00–5.00]	5.00 [5.00–5.00]	0.408
IA-2 (IU/mL)	10.00 [10.00–10.00]	10.00 [10.00–10.00]	0.629
ZnT8 (RU/mL)	10.00 [10.00–10.00]	10.00 [10.00–10.00]	0.604
Insulinemia (mU/L)	14.50 [11.75–19.00]	2.00 [2.00–2.00]	0.172
C-Peptide (ng/mL)	1.27 [0.81–1.67]	1.29 [1.12–1.93]	0.533
Glycemia (mg/dL)	77.00 [72.00–80.00]	73.00 [72.00–76.00]	0.341
IL-2 (pg/mL)	6.18 [0.00–6.97]	3.51 [0.00–7.08]	0.935
IL-4 (pg/mL)	1.88 [1.40–2.54]	1.98 [0.99–3.08]	1.000
IL-6 (pg/mL)	2.22 [1.60–5.27]	2.23 [1.00–3.78]	0.429
IL-8 (pg/mL)	37.71 [27.92–54.37]	14.54 [9.16–43.48]	0.102
IL-10 (pg/mL)	1.10 [0.77–1.39]	1.24 [0.85–1.66]	0.492
VEGF (pg/mL)	114.31 [72.48–247.77]	209.70 [95.57–277.23]	0.235
INF-γ (pg/mL)	1.50 [0.00–3.92]	0.00 [0.00–2.25]	0.382
TNF-α (pg/mL)	2.67 [2.11–4.31]	2.51 [2.01–2.64]	0.398
IL-1α (pg/mL)	0.72 [0.65–1.04]	0.82 [0.49–0.96]	0.916
IL-1β (pg/mL)	2.04 [1.73–3.66]	2.37 [1.49–3.46]	1.000
MCP-1 (pg/mL)	197.82 [165.28–245.61]	199.98 [154.94–219.48]	0.654
EGF (pg/mL)	149.61 [97.16–159.41]	141.63 [112.87–152.50]	0.692
Infectious diseases	0 (0.0%)	0 (0.0%)	1.000
Congenital hypothyroidism/endocrine disease	0 (0.0%)	1 (2.9%)	0.333
Allergies	0 (0.0%)	0 (0.0%)	1.000
Genetic syndromes	0 (0.0%)	1 (2.9%)	0.333
Celiac disease	68 (100.0%)	34 (100.0%)	1.000
Anti-TPO positivity	0 (0.0%)	1 (2.9%)	0.333
Anti-Tg positivity	1 (1.5%)	0 (0.0%)	1.000
Islet autoantibody positivity (GAD, IA-2 or ZnT8) ^‡^	4/64 (6.3%)	0/11 (0.0%)	0.298
Sex (male)	21 (30.9%)	7 (20.6%)	0.388

^‡^ Valid percentages for islet autoantibody positivity were calculated using the number of patients tested for type 1 diabetes-related autoantibodies (GAD, IA-2, or ZnT8) as the denominator. * denotes significant results.

**Table 4:** Cytokine levels by sex in celiac patients.

Variable	Females (*n* = 21)	Males (*n* = 7)	*p*-Value
IL-2 (pg/mL)	6.02 [3.45–7.40]	0.00 [0.00–0.00]	0.002 *
IL-4 (pg/mL)	2.34 [1.60–4.06]	1.27 [1.08–1.48]	0.046 *
IL-6 (pg/mL)	2.93 [1.45–5.68]	2.05 [0.99–3.78]	0.367
IL-8 (pg/mL)	18.36 [12.66–44.62]	16.78 [16.21–42.58]	1.000
IL-10 (pg/mL)	1.25 [0.87–1.45]	0.77 [0.39–0.87]	0.035 *
VEGF (pg/mL)	186.69 [82.94–277.23]	173.79 [76.27–202.99]	0.595
INF-γ (pg/mL)	1.86 [0.00–3.11]	0.00 [0.00–0.57]	0.327
TNF-α (pg/mL)	2.57 [2.05–2.78]	2.29 [2.08–3.62]	0.873
IL-1α (pg/mL)	0.84 [0.64–1.22]	0.63 [0.50–0.72]	0.059
IL-1β (pg/mL)	2.54 [1.85–3.48]	1.73 [1.23–2.67]	0.144
MCP-1 (pg/mL)	206.48 [161.07–232.07]	213.51 [177.97–266.92]	0.339
EGF (pg/mL)	141.63 [111.92–148.24]	158.87 [72.65–172.18]	0.633

* denotes significant results.

**Table 5:** Autoantibody titers by sex in celiac patients with available cytokine measurements.

Variable	Females (*n* = 21)	Males (*n* = 7)	*p*-Value
Anti-TPO (U/mL)	37.90 [30.50–39.20]	33.60 [31.85–40.05]	0.873
Anti-Tg (IU/mL)	1.30 [1.30–1.30]	1.30 [1.30–1.30]	0.621
tTG-IgA (IU/mL)	56.00 [1.00–128.00]	0.30 [0.20–0.90]	0.051

## Data Availability

The original contributions presented in this study are included in the article. Further inquiries can be directed to the corresponding authors.
